# Correspondence

**Published:** 1859-07

**Authors:** 


### Correspondence.

LOUISVILLE, Ky., June 14, 1859.

J. T. TOLAND, Esq.—Dear Sir: At your solicitation, on a very short notice, I send for your disposal a few sentences on the subject of Amalgam as used in mechanical dentistry. Perhaps a less objectionable name, amalgam, should be chosen for the "New Method of Mounting Teeth," as, to many, that much abused material savors of charlatanism, particularly when used IN A DENTAL sense. There certainly IS something in a name, Shakspeare to the contrary notwithstanding.

Crystal silver work! sounds very silvery. Crystal gold *mounting!* would be a high-sounding name, and though suggestive of Pike's Peak, might not be objectionable.

But to the point, and to relieve the minds of the anti-amalgam readers, let their prejudice on that head evaporate into "thin air," as is the mercury *evaporated* before the work goes into the head of the patient. To be brief, I shall simply give the process as I have practiced it, that others who have an eye to the so-called improvement, may be better able to judge of its probable merits, and to give it a trial for themselves. The plates are to be swaged up, in the usual way, and if the ordinary gum teeth are used, grind and back them as if to be soldered. While encased in plaster, score (roughen) the linings of the teeth and the plate to the extent necessary,—wash with alcohol and brush, to remove all grease, wax, or dirt; apply a thin coating of mercury to the roughened surface, to insure cohesion, before working on the amalgam, which should be applied in as *dry* a state as possible, for, in proportion to the quantity of mercury to be driven off, will the work be dense or porous.

The amalgam must be made with precipitated metallic silver and distilled mercury. It should be washed in a mortar with alcohol as long as the latter continues to be discolored. Very much depends on having pure materials and clean surfaces for this style of work. The material may be worked on with a plug-file and one or two other flat and pointed instruments for working in close places. Slope the amalgam on the palatine surface, producing as natural form as the case will allow. Build up a little beyond where you want the work when finished, as, after the mercury is driven off, the crystals are susceptible of being *condensed.* A finish over the top of the gums may be made with this material which, in point of beauty, surpasses any other style of "riming." It is for this purpose that I have used it more than any other. I always doubted its strength to withstand the pressure of mastication; I am now testing it, however, in this way.

The work moulded, the next step is, to dispel the mercury by the application of heat. This must be *slowly* and gradually applied—and the temperature should not be raised above two or three hundred degrees. To protect the teeth, suspend the case of work in a tin or sheet-iron cylinder, large enough in diameter to receive it, and twelve or fifteen inches in length. Place a small spirit-lamp below—leaving sufficient opening above to allow the fumes of mercury to escape. From one to three hours' time should be allowed for the evaporating process.

The vessel should be set in a flue or where the vapor will be carried away, as it would be deleterious to inhale.

The work cool, proceed to condense, file, rub down and finish as you would a crystal gold plug.

The work presents beauty and apparent strength, but its solidity is only superficial. It is my opinion that in time, the fluids of the mouth will find their way through the more porous body, and cause it to become rancid and brittle, like the interior of the first sponge gold-fillings that were made.

In addition, I can only say that some *riming* which I did in this style, both with gold and silver, some two months ago, still looks well. I hope some others who, perhaps, have more time than myself to experiment in this direction, will give it a trial, and report. J. A. McClelland.

### A SUGGESTION.

MESSRS. EDITORS: Seeing "a call" for a "convention" to form a "National Association of Dentists," a thought has occurred to my mind which may be of some importance to those interested in the formation of this new "association," and with your permission, I would here offer it to the con- ideration of all those who may be "selected" "to go" to this important meeting.

The suggestion is: That the "delegates" to this convention, choose, as the only fit personage to preside over the destinies of the "National Association of Dentists" in the United States of America, his excellency the Grand Duke of *Tusc*-any. *Any* other *Tusk*-doctor would scarcely be so fit as the *grand duke.* And if the Emperor of Austria can spare his excellency, and whip *Ec. paron Louis!* without him, every effort should be made to get the duke over in time for this new conventien. He certanly would purge the association of its "*democratic*" element, and place it at once on a high and honorable basis.

I remain yours, very truly, BOURBON.

